# Prevalence and associated factors of neonatal hypothermia in the neonatal intensive care unit at St. Peter’s specialized Hospital, Ethiopia

**DOI:** 10.1038/s41598-025-34527-w

**Published:** 2026-01-14

**Authors:** Etalemahu Sebro, Tseganesh Asefa, Abraham Eshetu Mamo, Million Molla Sisay, Altaye Nigussie, Eyerusalem Mekonnen, Workye Tigabie Molla, Esubalew Amsalu Tibebu, Endalkchew Biranu

**Affiliations:** 1 Research, and Evidence Generation Directorate, St Peter’s Specialized Hospital, Addis Ababa, Ethiopia; 2 Clinical Trial Directorate, Armauer Hansen Research Institute, Addis Ababa, Ethiopia

**Keywords:** Ethiopia, Hypothermia, Neonate, NICU, Risk factors, Health care, Medical research, Risk factors, Signs and symptoms

## Abstract

Neonatal hypothermia, defined as a body temperature below 36.5 °C, is a global issue that significantly increases neonatal morbidity and mortality, particularly in low- and middle-income nations. Despite global guidelines, the prevalence remains high in many resource-limited settings. This study aimed to assess the prevalence and associated factors of hypothermia among neonates admitted to the intensive care unit of St. Peter’s Specialized Hospital. An institution-based cross-sectional study was conducted from July to September 2024 among 222 neonates admitted to the intensive care unit of St Peter’s Specialized Hospital. The data were collected using a semi-structured questionnaire and chart review. Binary and multivariate logistic regression analyses were used to identify factors associated with hypothermia at a significance level of *p* < 0.05 using Statistical Package for Social Sciences (SPSS) version 25.0 software. Prevalence of neonatal hypothermia was 66.7%. Neonates from families earning ≤ $100 (AOR = 2.60, 95% CI: 1.25, 5.42), aged ≤ 24 h (AOR = 2.85, 95% CI: 1.85, 5.05), with a gestational age < 37 weeks (AOR = 4.50, 95% CI: 2.40, 6.50), with a lack of thermal care (AOR = 2.75, 95% CI: 1.50, 4.40), with improper wrapping at admission (AOR = 5.50, 95% CI: 3.60, 9.80), and with inborn delivery (AOR = 2.75, 95% CI: 1.45, 5.00) increased the odds of hypothermia. However, single pregnancy (AOR = 0.36, 95% CI: 0.15, 0.88) decreases the odds of hypothermia. Neonatal hypothermia remains highly prevalent among neonates admitted to the intensive care unit of St. Peter’s Specialized Hospital. Significant risk factors were low income, neonates aged ≤ 24 h, gestational age < 37 weeks, lack of thermal care, improper wrapping at admission, and inborn delivery. While a single pregnancy is a proactive factor. Adherence to thermal care practices guidelines, particularly during the first hours of life, is crucial. Healthcare facilities should enforce policies, provide regular training, and evaluate the effectiveness of national and World Health Organization guidelines.

## Introduction

Neonatal hypothermia is defined by the World Health Organization (WHO) as an abnormal thermal state in which the body temperature is below 36.5 °C (97.7^o^F) among newborns under 28 days of age^1^. Neonates are particularly vulnerable to hypothermia due to their large surface area-to-body mass ratio, decreased subcutaneous tissue, high body water content, immature metabolic mechanisms, and poorly regulated skin blood flow^2,3^. They lose heat through conduction, convection, radiation, and evaporation, especially immediately after birth when they transition from the warm intrauterine environment to the external world^4^.

Hypothermic neonates are categorized as mild (36.0˚C-36.4˚C), moderate (32.0˚C-35.9˚C), and severe (< 32.0˚C), with increasing severity associated with worse outcomes^1,5^. Hypothermic neonates are susceptible to complications such as peripheral vasoconstriction, reduced peripheral perfusion, ischemia, metabolic acidosis, and elevated basal metabolic rate. Furthermore, the condition can worsen respiratory distress and predispose neonates to pulmonary hemorrhage and disseminated intravascular coagulation^5–7^.

Globally, neonatal hypothermia is a significant contributor to neonatal morbidity and mortality, especially in low- and middle-income countries (LMICs)^8,9^. Although it is often underreported, hypothermia is a common occurrence, particularly among neonates born at home without appropriate thermal care. A high incidence of primary hypothermia has been documented within the first 24 h of life. Despite advances in neonatal care, studies from technologically advanced neonatal intensive care units (NICUs) report hypothermia incidence rates ranging from 31% to 90% among very low birth weight neonates on admission^8,10,11^.

According to UNICEF, 2.3 million children died in the first month of life in 2021—approximately 6,400 neonatal deaths each day. More than 99% of these deaths occur in developing countries, where the average neonatal mortality rate is about 33 per 1,000 live births, compared to 4 per 1,000 in high-income countries^12,13^. Notably, over 40% of under-five mortality occurs during the neonatal period. Sub-Saharan Africa continues to bear the highest burden, with an under-five mortality rate of 74 per 1,000 live births—15 times higher than in Europe and Northern America^14,15^.

Although hypothermia may not always be the direct cause of death, it contributes significantly as a comorbid condition, undermining newborn survival. Maintaining normothermia is therefore essential in neonatal care^6,16^. Therapeutic hypothermia has proven beneficial in specific medical conditions, however, the unintentional and unintended drop in body temperature remains a pressing issue^17,18^. Despite these clinical advances, national and WHO recommendations for neonatal thermal care are not consistently implemented, as studies highlight systemic and facility-level barriers that impede adherence. These include nonfunctional warming equipment, supply shortages, weak infrastructure, inadequate staff training. infrastructure and weaken referral system^19,20^. Therefore, this study aimed to assess the current prevalence and associated factors of neonatal hypothermia among neonates admitted to NICUs of St. Peter Specialized Hospital.

## Materials and methods

### Study design, period, and setting

An institution-based cross-sectional study was conducted from July to September 2024 at the NICU of St. Peter’s Specialized Hospital, Addis Ababa, Ethiopia. St. Peter’s Hospital was established in 1953. It is administered under the Ethiopia Federal Ministry of Health. The hospital has delivered multi-service as a general hospital with a workforce of over 1500 administrative and technical personnel; the hospital has provided a variety of services as a general hospital, including the NICU and maternal and child unit (MCH).

### Study population

All neonates aged less than 28 days who were admitted to the NICU at St. Peter Specialized Hospital during the study period, along with their mothers, were included in the study. Neonates with incomplete medical records and those with congenital anomalies incompatible with life were excluded.

### Sample size determination and sampling technique

To determine the sample size, a single population proportion formula was used.

n =$\:\:\:\frac{(\mathrm{Z}\:{\upalpha\:}\:/2)2\:\mathrm{P}\:(1-\mathrm{P})}{\mathrm{d}2}$, the proportion of hypothermia in neonates is 83.2% from a previous study^18^, assuming a 95% confidence level (1.96) and a 5% margin of error as follows:

n = $$\:\frac{\left(1.96\right)2\:0.168(1-0.832)}{\left(0.05\right)2}$$ = 214.

Where n = required sample size; z = critical value at 95% CI; p = prevalence rate; d = margin of error to be 5%.

After adding a 10% nonresponse rate, the final sample size was 235.

A systematic random sampling technique was used to approach the study participants at the NICU. The sampling interval was calculated via the formula k = $\:\frac{Nt}{n}$.

Where Nt = the estimated total population size over a three-month period based on hospital records is 281, n = the total sample size, and k = 281/235 = 1.19 ≈ 1.

Participants were selected subsequently until the desired sample size was reached.

### Variables

#### Dependent variable

Neonatal hypothermia.

#### Independent variables

Socio-demographic and obstetric characteristics such as age, region, religion, residence, educational status, occupation, income, pregnancy type, mode of delivery, parity, ANC follow-up, complications, and type of obstetric complications.

Neonatal and environmental factors included the neonate’s age in hours, sex, birth weight, gestational age, bath within the first 24 h, skin-to-skin contact, drying after birth, early initiation of breastfeeding, warm transport, cardiopulmonary resuscitation (CPR) at birth, proper wrapping at admission, delivery site, delivery room temperature, deliver site, delivery time, NICU admission time, and NICU room temperature.

### Operational definition

- **Hypothermia: **An axillary temperature of less than 36.5 °C.
- **Mild hypothermia (cold stress): **An axillary temperature of 36.0–36.4 °C.
- **Moderate hypothermia: **An axillary temperature of 32.0 °C to 35.9 °C.
- **Severe hypothermia:** An axillary temperature of < 32.0 °C.
- **Non-hypothermic: **An axillary temperature of ≥ 36.5 °C.
- **Neonate: **An infant under 28 days of age.
- **Inborn: **Neonates born in the study hospital (St. Peter Specialized Hospital).
- **Outborn: **Neonates born outside the study hospital.
- **Admission temperature: **The first temperature obtained from neonates at admission to NICU.
- **Thermal care**: A composite of three essential neonate practices: immediate post-birth drying, skin-to-skin contact, and early breastfeeding: Neonates were classified as “Yes” if all three practices were performed and “No” if any of the practices were not performed.

### Data collection tools and procedures

The eligible study participants were approached, and they provided informed written consent regarding their willingness to participate in the study^7,21–23^. An interview followed. The information was gathered via a structured interviewer-administered questionnaire and a chart review technique. The questionnaire was adopted from previous studies^21–23^. First, it was developed in English and then translated into the Amharic version and back-translated by the bilingual speaker to ensure consistency. The axillary temperature of the neonate was measured at the point of admission by using a digital thermometer. The data was collected by two BSc neonatal nurses who were working in the NICU and supervised by one MSc NICU nurse professional.

### Data quality control

To ensure the quality of the data, the recruited data collectors and supervisors underwent training sessions lasting half a day. They were trained on the objective, confidentiality of information, relevance of the study, respondents’ rights, informed consent, and interview techniques. The questionnaire was pretested on 5% of the final sample size at Yekatit 12 Hospital. Regular supervision was taken during the data collection period to check the data completeness. Furthermore, the tool was tested for internal consistency (reliability), and a Cronbach’s alpha of α = 0.84 was obtained.

### Data processing and analysis

The data was cleaned, coded, and entered into Epi info version 3.1 and exported to SPSS version 25 software for further analysis. Frequency distribution and percentage of variables were computed to describe and summarize the data in tables and graphs. A bivariate binary logistic regression was used to identify variables at p-value < 0.25. Finally, the independent predictors were identified by using multivariate logistic regressions. The cut point to declare the presence of an association was a p-value < 0.05.

## Result

### Socio-demographic and obstetric characteristics of mothers of neonates

A total of 235 neonates were included in the study with a 94.5% response rate. The participants’ mothers’ ages ranged from 18 to 47 years, and the mean age of mothers was 28 ± 5.2 years, and over half of the mothers, 116 (52.3%), were aged between 21 and 29 years. One hundred sixty-five (74.3%) came from the Addis Ababa region. Two hundred two (91.0%) were urban residents. Sixty-six respondents (29.7%) were primary school, and 132 (59.4%) of respondents were housewives. The monthly income of the family was below ≤$100 for 120 (54.1%) of participants. Most neonates’ mothers (188, 84.7%) delivered without obstetric complications, and the pregnancies were predominantly single-birth. More than half, 135 (60.8%), were delivered through spontaneous vaginal delivery. Two hundred sixteen (97.3%) of the mothers had visited health facilities for antenatal care during the recent pregnancy (Table 1).

**Table 1:** Socio-demographic and obstetric characteristics of mothers of neonates admitted to the NICU at St. Peter specialized hospital (*n* = 222).

Variables	Categories	Frequency	Percentage (%)
Age (year)	≤ 20	20	9.0
	21–29	116	52.3
	> 29	86	38.7
	Mean ± SD	28 ± 5.2	
Region	Addis Ababa	165	74.3
	Oromo	55	24.8
	Amhara	2	0.9
Religion	Orthodox	185	83.3
	Muslim	16	7.2
	Protestant	21	9.5
Residence	Urban	202	91.0
	Rural	20	9.0
Educational status	No formal education	24	10.8
	Primary school	66	29.7
	Secondary school	62	28.0
	Diploma	24	10.8
	Degree and above	46	20.7
Occupation	Housewife	132	59.4
	Government employees	41	18.5
	Private and others	49	22.1
Monthly income of the family	≤$100	120	54.1
	$101 – $200	65	29.2
	≥$201	37	16.7
Obstetric complications during pregnancy	No	188	84.7
	Yes	34	15.3
Kind of obstetric complication During pregnancy	Hypertension	28	82.4
	Isolated thrombocytopenia	1	2.90
	Premature rupture of membrane	5	14.7
Pregnancy type	Single	188	84.7
	Twin	32	14.4
	Triple	2	0.90
Mode of delivery	C/S	84	37.8
	Instrumental	3	1.40
	Spontaneous vaginal delivery	135	60.8
Parity	Multiparous	122	55.0
	Primiparous	100	45.0
ANC follow-up during the last pregnancy	No	6	2.7
	Yes	216	97.3
Number of ANC follow-ups	< 4	33	14.9
	≥ 4	189	85.1

### Neonatal and environmental characteristics of neonates admitted to the NICU at St. Peter specialized hospital

More than half of neonates were males (61.3%) and aged ≤ 24 h (62.6%) at the time of data collection. Similarly, more than half of the neonates had a birth weight ≥ 2500 g (62.2%), with a mean birth weight of 2682.77 ± 781 g. About two-thirds (67.6%) were born at term gestation (≥ 37 weeks), with a mean gestational age of 37 ± 3.3 weeks.

Regarding thermal care practices, 96.8% of neonates were not bathed within the first 24 h, 47.3% had skin-to-skin contact, and 77.5% were dried immediately after birth. However, only 38.7% were early initiations of breastfeeding. Warm transport was practiced for 65.3%, and 8.6% required CPR at birth. At admission, 88.3% were properly wrapped.

In terms of delivery characteristics, 62.6% of neonates were inborn deliveries; among them, 56.1% were delivered at a delivery room temperature of < 22 °C. Among outborn deliveries, 54.2% occurred in health centers, 32.5% in other hospitals, and 9.6% at home. Daytime deliveries and NICU admissions accounted for 52.3% and 73%, respectively. About three-fourths (74.8%) of neonates were admitted to the NICU room with a temperature below 22 °C (Table 2).

**Table 2:** Neonatal and environmental characteristics of neonates admitted to NICU at St. Peter specialized hospital (*n* = 222).

Variables	Categories	Frequency	Percentage (%)
Age of neonate	≤ 24 h	139	62.6
	> 24 h	83	37.4
Sex of neonate	Female	86	38.7
	Male	136	61.3
Birth weight	< 2500 g	84	37.8
	≥ 2500 g	138	62.2
	Mean ± SD	2682.77 ± 781 g	
Gestational age	< 37 weeks	72	32.4
	≥ 37 weeks	150	67.6
	Mean ± SD	37 ± 3.3 weeks	
Bath within the first 24 h	No	215	96.8
	Yes	7	3.20
Skin-to-skin contact	No	117	52.7
	Yes	105	47.3
Drying after birth	No	50	22.5
	Yes	172	77.5
Early initiation of breastfeeding	No	136	61.3
	Yes	86	38.7
Warm transport (intra-/extra-facility)	No	77	34.7
	Yes	145	65.3
CPR at birth	No	203	91.4
	Yes	19	8.60
Proper wrapping at admission	Well covered	196	88.3
	Not well covered	26	11.7
Delivery site	Inborn	139	62.6
	Outborn	83	37.4
Delivery room temperature	< 22 °C	78	56.1
	≥ 22 °C	61	43.9
Outborn delivery site	Health center	45	54.2
	Homes	8	9.60
	Other hospitals	27	32.5
	Private health facility	2	2.40
	Traditional birth center	1	1.3
Delivery time	Day	116	52.3
	Night	106	47.7
NICU admission time	Day	162	73.0
	Night	60	27.0
NICU room temperature	< 22 °C	170	76.6
	≥ 22 °C	52	23.4

### Medical diagnosis of the neonates

Medical diagnoses during admission were reviewed from the medical record of the neonates, and 46 (16.3%) were admitted for the reason of sepsis, 44 (15.5%) as diagnoses of prematurity, 33 (11.7%) were diagnosed with hypothermia, 27 (9.5%) were admitted for the reason of respiratory distress, 27 (9.5%) were diagnosed as low birth weight, 23 (8.2%) were diagnosed as failure of suck, and 15 (5.3%) were diagnosed with neonatal jaundice (Table 3).

**Table 3:** Neonatal diagnoses at NICU admission, St. Peter specialized Hospital.

Variable	Categories	Frequency	Percentage (%)
Diagnosis during admission	LBW	27	9.5
	Hypothermia	33	11.7
	Prematurity	44	15.5
	Sepsis	46	16.3
	Respiratory distress	27	9.5
	Failure of suck	23	8.2
	Jaundice	15	5.3
	LPT	12	4.3
	AGA	14	4.9
	Intrauterine growth retardation	15	5.3
	Other	27	9.5

The total frequency exceeds the sample size because some neonates had two or more diagnoses at admission.

### Prevalence of neonatal hypothermia among neonates admitted to NICU at St. Peter specialized hospital

**Fig. 1:**
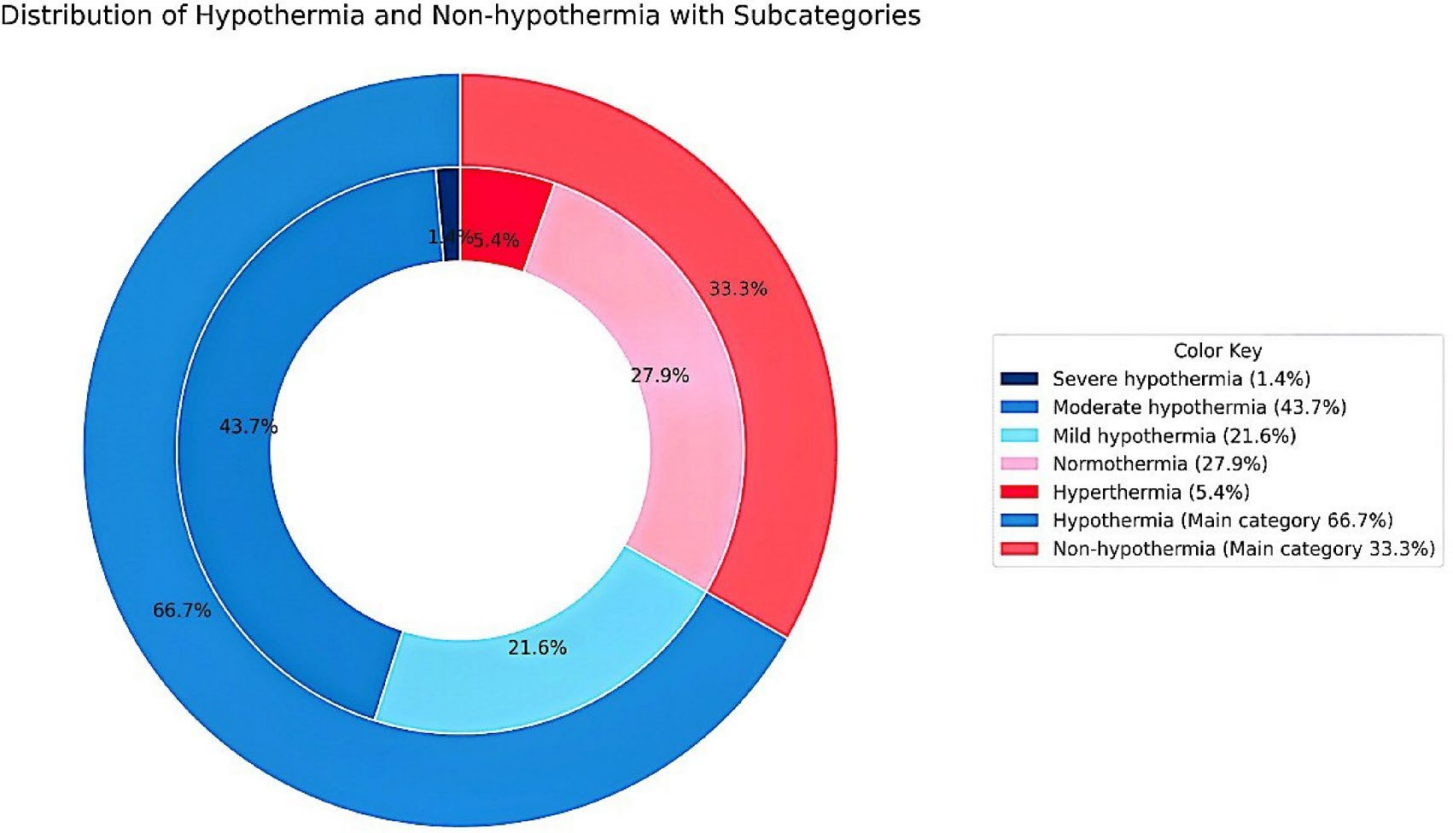
Proportion of hypothermia and its subcategories among neonates admitted to the NICU at St. Peter Specialized Hospital.

The prevalence of neonatal hypothermia is 66.7% (95% CI: 60.4% − 73%). The outer pie chart shows the overall distribution of hypothermia 148 (66.7%) and non-hypothermia 74 (33.3%) cases. The inner nested charts illustrate the subcategories: among hypothermic neonates, severe hypothermia accounted for 3 (1.4%), moderate hypothermia 97 (43.7%), and mild hypothermia 48 (21.6%); among non-hypothermic neonates, normothermia represented 62 (27.9%) and hyperthermia 12 (5.4%) (Fig. 1).

### Factors associated with neonatal hypothermia

In bivariate logistic regression analysis, variables with *p* < 0.25 were included in the multivariable analysis. From the twelve variables included in the multivariable analysis, seven variables were statistically significant at *p* < 0.05. Accordingly, income, neonates aged ≤ 24 h, gestational age < 37 weeks, lack of thermal care, improper wrapping, single pregnancy, and inborn delivery were demonstrated to have statistically significant associations with hypothermia.

Neonates from families earning ≤ $100 monthly were 2.60 times more likely to develop hypothermia (AOR = 2.60, 95% CI: 1.25, 5.42). Neonates aged ≤ 24 h were almost three times more likely to have hypothermia (AOR = 2.85, 95% CI: 1.85, 5.05).

Preterm neonates with a gestational age of < 37 weeks had over 4 times higher odds of hypothermia (AOR = 4.50, 95% CI: 2.40, 6.50). Neonates who did not receive proper thermal care were 2.75 times more likely to develop hypothermia (AOR = 2.75, 95% CI: 1.50, 4.40). Lack of proper wrapping at the time of admission made them almost six times more likely to develop hypothermia (AOR = 5.50, 95% CI: 3.60, 9.80). Neonates from single pregnancies were 64% less likely to develop hypothermia (AOR = 0.36, 95% CI: 0.15, 0.88). Neonates delivered within the facility were 2.75 times more likely to develop hypothermia (AOR = 2.75, 95% CI: 1.45, 5.00) (Table 4).

**Table 4:** Bivariate and multivariate logistic regression analysis of factors associated with neonatal hypothermia among neonates admitted to NICU at St. Peter specialized Hospital.

Variable	Category	COR (95% CI)	*p*-value	AOR (95% CI)	*p*-value
Monthly income of the family	≤ $100	1.45 [1.10, 1.92]	0.008	2.60 [1.25, 5.42]	0.012*
	$101–$200	1.10 [0.68, 1.79]	0.695	2.10 [1.07, 4.42]	0.051
	≥ $201	1	1	1	
Age of neonates	≤ 24 h	1.40 [1.05, 1.88]	0.020	2.85 [1.85, 5.05]	0.011*
	> 24 h	1		1	
Sex of neonates	Female	0.90 [0.60, 1.34]	0.062	0.80 [0.45, 1.45]	0.053
	Male	1		1	
Birth weight	< 2500 g	1.55 [1.05, 2.29]	0.028	1.10 [0.45, 2.62]	0.180
	≥ 2500 g	1		1	
Gestational age	< 37 weeks	2.65 [1.75, 4.02]	0.021	4.50 [2.40, 6.50]	0.011*
	≥ 37 weeks	1		1	
Thermal care	No	3.20 [2.05, 4.99]	0.001	2.75 [1.50–4.40]	0.019*
	Yes	1		1	
CPR at birth	No	1.25 [0.55, 2.85]	0.085	0.55 [0.18, 1.60]	0.248
	Yes	1 °C		1	
Proper wrapping at admission	Not well covered	3.95 [2.90, 6.20]	0.003	5.50 [3.60, 9.80]	0.006*
	Well covered	1		1	
Pregnancy type	Single	0.90 [0.42, 1.95]	0.191	0.36 [0.15, 0.88]	0.025*
	Multiple	1		1	
Delivery site	Inborn	1.95 [1.20, 3.15]	0.007	2.75 [1.45, 5.00]	0.004*
	Outborn	1		1	
Delivery time	Day	1.45 [0.90, 2.34]	0.125	1.50 [0.78, 2.85]	0.232
	Night	1		1	
NICU room temperature	< 22 °C	1.60 [0.95, 2.70]	0.075	1.38 [0.76, 2.50]	0.255
	> 22 °C	1		1	

*Indicates statistically significant association at *p* < 0.05.

## Discussion

In this study, the overall result showed that the prevalence of neonatal hypothermia is 66.7% among neonates admitted to the NICU, with most cases being moderate in severity. The magnitude is consistent with findings from studies conducted in Dessie, Ethiopia (66.8%)^6^, and Saudi Arabia (66%)^24^, indicating the persistence of hypothermia as a serious neonatal health issue in various settings. However, it is slightly lower than the prevalence reported in Northern Nigeria (72.9%)^25^, Zimbabwe (85%)^26^, Babol, Iran (84.5%)^27^, and Uganda (83%)^21^. This variation might be due to differences in clinical protocols, climate conditions, and infrastructure such as thermal protocols and neonatal warmers. Conversely, lower prevalence rates were reported in East Africa (57.2%)^28^, South Africa (21%), and Tanzania (22%)^29^. This might be attributed to improved early thermal care practices, the use of kangaroo mother care, and more rigorous adherence to WHO guidelines.

Our findings indicate that neonates from families with lower monthly income were more likely to develop hypothermia. This association may reflect limited access to thermal care, adequate clothing, or heating in low-income households, highlighting the impact of socioeconomic status on neonatal outcomes. These findings are consistent with previous studies in Nepal^30,31^, which found a higher incidence of hypothermia among infants from families with ‘below average’ income. Similarly, a study in low socioeconomic status among determinants of neonatal hypothermia^32^.

Neonate age was another critical factor; neonates aged ≤ 24 h were significantly more likely to develop hypothermia. Which is consistent with findings from Ethiopia^17^ and Bangladesh^31^. This could be due to the fact that the vulnerability of neonates in the first hours of life is due to their limited ability to regulate body temperature.

Gestational age also played a significant role, with preterm neonates (< 37 weeks) being significantly more likely to develop hypothermia. This finding is similar to a study done in Ethiopia^33^, East Africa^28^, Pakistan^34^, and Iran^27^. The possible reason for this finding might be preterm neonates have a large surface area-to-body mass, minimal subcutaneous fat stores, poor clinical status, low tone, limited capacity to generate heat from fat stores, and immature and thin skin that increases heat loss through radiation^6,35,36^.

Lack of thermal care was significantly associated with neonatal hypothermia. This finding aligns with studies conducted in Ethiopia^24^, Ghana^37^, and South Asia^38^. This could be due to essential thermal care practices, such as prompt drying, skin-to-skin contact, and early initiation of breastfeeding. Prompt drying reduces evaporative heat loss immediately after birth, skin-to-skin contact allows the newborn to gain heat through conduction from the mother, and early breastfeeding provides an immediate energy source necessary for thermogenesis. Collectively, these practices help maintain the neonate’s body temperature and reduce the risk of hypothermia^33,39^.

In this study, neonates who had not been properly wrapped during admission had increased odds of hypothermia. This finding was similar to the research conducted in Ethiopia^40^ and Kenya^4^. This could be due to the fact that the neonates’ huge skulls, which have open fontanels and sutures, contribute to nearly 25% of heat loss if not covered by cloth.

Moreover, this study showed that neonates from single pregnancies were significantly less likely to develop hypothermia. This result is supported by studies conducted in Eastern Maharashtra, India^41^, and Ethiopia^42^. This protective effect may be attributed to the increased clinical attention and individualized care typically given during single births. In contrast, multiple births often present challenges, including preterm delivery, low birth weight, and shared resources (such as warmth or maternal contact), all of which can increase the risk of thermal instability.

Lastly, this study showed that inborn delivery was associated with a significantly higher risk of neonatal hypothermia compared to outborn delivery. This finding aligns with studies conducted in Ghana^11^. This might be due to cold delivery rooms, lack of warmers, delayed initiation of skin-to-skin contact, and postponed breastfeeding disrupting the neonatal “warm chain,” thereby increasing the risk of hypothermia in newborns^33,43^.

The high prevalence of neonatal hypothermia identified in this study underscores the urgent need for practical strategies to improve adherence to thermal-care guidelines. Evidence suggests that sustainable improvement requires a structured quality improvement approach that integrates three key components. First, ongoing monitoring and feedback mechanisms, such as temperature-monitoring indicators, dashboards, and regular audits, enable early identification of gaps in thermal care practices. Second, administrative commitment and team-based engagement foster a culture of accountability, ensuring that thermal care is prioritized as a routine component of neonatal management. Third, staff education and empowerment, including competency-based training and ensuring the availability and functionality of warming equipment, supports consistent application of guidelines. Implementing these strategies can enhance adherence to WHO and national recommendations, ultimately reducing the burden of neonatal hypothermia and improving neonatal outcomes.

### Conclusions and recommendations

Neonatal hypothermia remains one of the significant health concerns among neonates admitted to the NICU of St. Peter’s Specialized Hospital. Significant risk factors were low income, neonates aged ≤ 24 h, gestational age < 37 weeks, lack of thermal care, improper wrapping at admission, and inborn delivery. While single pregnancy is a proactive factor.

To overcome this long-standing problem, it is crucial to strengthen compliance with the WHO thermal care guidelines, especially during the first hours of life. This includes immediate drying, skin-to-skin contact, breastfeeding, and proper wrapping. Beyond policy, a structured quality improvement strategy is recommended, integrating: (1) ongoing monitoring with clear indicators and dashboards, (2) administrative and team-based engagement to promote a culture of accountability, and (3) continuous staff education, training, and access to functional warming equipment.

Healthcare facilities should enforce policies for optimal thermal care, and regular training and mentorship programs are crucial. Community education and increased financial support can also reduce the risk of hypothermia and improve neonatal survival. Future research should focus on evaluating the implementation, adherence, and effectiveness of national and WHO guidelines to identify and address gaps in maternal-neonatal care practices to reduce neonatal hypothermia.

## Data Availability

The data will be available from the corresponding author upon reasonable request.
